# Evaluating patients’, physicians’ and pharmacy professionals’ perception and concern regarding generic medicines in Gondar town, northwest Ethiopia: A multi-stakeholder, cross-sectional survey

**DOI:** 10.1371/journal.pone.0204146

**Published:** 2018-11-07

**Authors:** Begashaw Melaku Gebresillassie, Sewunet Admasu Belachew, Yonas Getaye Tefera, Tamrat Befekadu Abebe, Abebe Basazn Mekuria, Kaleab Taye Haile, Daniel Asfaw Erku

**Affiliations:** 1 Clinical Pharmacy Departments, School of Pharmacy, College of Medicine and Health Sciences, University of Gondar, Gondar, Ethiopia; 2 Pharmacology Department, School of Pharmacy, College of Medicine and Health Sciences, University of Gondar, Gondar, Ethiopia; 3 Pharmaceutics and Social Pharmacy Department, School of Pharmacy, College of Medicine and Health Sciences, University of Gondar, Gondar, Ethiopia; Newcastle University, UNITED KINGDOM

## Abstract

**Background:**

Generic medicine prescribing has become common practice in many countries. However, data regarding the perceptions of stakeholders (patients, prescribers and dispensers) regarding generic medicines in Ethiopia is scarce. The present survey aimed to investigate the perception of patients, physicians and pharmacy professionals regarding generic medicines in Ethiopia.

**Methods:**

A quantitative cross-sectional survey was conducted in Gondar town, Northwest Ethiopia between January 1 and February 1, 2017. The questionnaire, comprised of 5-point Likert scale items on perception and concerns regarding generic medicine, was administered to patients, physicians and pharmacy professionals working in the community drug retail outlets. Frequencies, percentages, and median were calculated using Statistical Package for the Social Sciences (SPSS) software version 21.0 for Windows to describe different variables.

**Results:**

The survey was completed by 612 participants. More than half of patients, 56% (n = 219) knew about the term brand and generic medicines and 38.7% (n = 151) of patients agreed/strongly agreed that brand medicines are more effective. Nearly half, 47% (n = 184) of patients agreed that they should have the option of choosing between generic and brand medicines and 61.4% (n = 240) of patients believed that cost should be considered before a medicineis prescribed. The majority of physicians 70.6% (n = 101) indicated a very low generic medicine prescription rate. 56% (n = 130) of physicians and 87.2% (n = 68) of pharmacy professionals agreed that they need a standard guideline to both prescribers and pharmacists on brand substitution process. Furthermore, 39.9% (n = 57) of the physicians and 87.2% (n = 68) of pharmacy professionals agreed that drug advertisements by the manufacturers would influence their prescribing/dispensing practice.

**Conclusions:**

Overall, our findings demonstrate a knowledge gap among patients towards the perception of generics, perceiving generics are less effective and inferior in quality compared to their branded equivalents. The majority of physicians reported a very low generic medicine prescribing rate and the majority pharmacy professionals’ dispensing practice was influenced by drug advertisements. Hence, a customized educational program should be developed and implemented to patients, prescribers and dispensers so as to boost the acceptability of generic medicines and increase generic prescribing and/or substitution.

## Background

With the rising global healthcare expenditure, governments and policy makers in many countries have implemented a number of cost-containment attempts so as to spend the limited financial resources in a more efficient and accessible way [1]. One of the strategies employed is the promotion of generic medicine use, which are generally, less expensive than their proprietary counterparts [2]. The World Health Organization (WHO) has defined generic medicine as “a pharmaceutical product that is usually intended to be interchangeable with an innovator product, is manufactured without a license from the innovator company and is marketed after the expiry date of the patent or other exclusive rights” [3]. Generic medicines exhibit an equivalent therapeutic effect when compared to branded medicines with a lowest price [4]. According to the findings from price surveys in 36 low and middle-income countries, the lowest cost of generic medicines in the private sectors were 2.6 times less expensive than their counterpart originator medicines [1]. As a result, the use of generic medicines is becoming a more popular way of reducing pressure on medicine budgets and they took an increasing percentage of overall dispensed medicines [5].

Many countries in the western world have introduced and advocated a variety of measures to increase the prescribing and dispensing of generic medicines [6–8] and resulted in significant cost savings, with more than $30 billion saved by European health care systems annually [9] and around $193 billion saved by the American health care organization in 2011 for using generic medicines [10]. A study conducted by the WHO in several developing countries, including African countries, estimated an average saving of up to 90% through generic substitution [11]. In Ethiopia, a six-fold cost reduction could be achieved by using generic equivalents rather than brand medicines [12].

The acceptance and utilization of generic medicines varies across different countries and is largely influenced by prescribers’, dispensers’ and patients’ perceptions, along with policy-related issues [13]. Recent systematic reviews on perceptions of generic medication in the general population, patients, doctors and pharmacy professionals identified that a substantial proportion of them hold negative perceptions of generic medicines, perceiving generics as less effective, less safe, inferior in quality and more likely to cause side effects compared to their branded equivalents [13, 14]. A study conducted in Germany also found that more than half of the patients hold negative views of generic medicines [15]; several other studies also reported similar findings [16–18]. Furthermore, the information given by a prescribing physician and/or dispenser on generic substitution is one of the contributing factors that influences patients’ perceptions about and acceptance of generic medicines [19–22].

The quality of generic medicines circulating in the market is also one of the main contributing factors for the negative views of different stakeholders, including prescribers and dispensers. According to the findings of a recent study conducted on quality assurance of medicines supplied to sub-Saharan African countries, pharmaceutical distributors active in these countries do not apply stringent criteria for selecting products and suppliers, which makes the product quality inconsistent and largely depends on the requirements of purchasers [23]. Two national surveys conducted in Ethiopia to evaluate the quality of marketed pharmaceuticals and its regulatory framework, reported that illegal medicines included both registered and unregistered products of which some were counterfeits and substandard. They were also reported that a substantial proportion of poor quality medicines such as: albendazole and mebendazole tablets were on the Ethiopian drug market, and from which one third of them (29%) were substandard [24, 25].

Data on stakeholders’ perceptions regarding generic substitution could help healthcare organizations, policy makers and other stakeholders to design and implement a robust generic medicine policy to promote the practice of generic substitution, as it is possible the negative perceptions of patients, prescribers or dispensers could represent a major barrier to the widespread adoption of generic medicines. This is especially true for sub-Saharan African countries like Ethiopia where access to quality affordable medicines is a major challenge and the main sources of drug expenditure is patients’ out of pocket money [26]. Although the importance of generic medicines on Ethiopian market is paramount in view of the limited financial resources for healthcare, there is still lack of data regarding the perception of stakeholders (patients, prescribers and dispensers) regarding generic medicines. The present survey was conducted with the aim of investigating the perceptions of patients, physicians and pharmacy professionals regarding generic medicines in Gondar, northwest Ethiopia.

## Materials and methods

### Study design and setting

A questionnaire-based quantitative cross-sectional study was conducted in Gondar town, northwest Ethiopia. Gondar town is located about 750 Km Northwest of Addis Ababa (the capital city of Ethiopia). According to the 2007 population and housing census report, the town had an estimated population of 206,987. The town has one referral and teaching hospital, 31 community pharmacies (run only by a pharmacist having a qualification of a university degree or above), 33 drug stores (run by druggist or pharmacy technician having a qualification of diploma in pharmacy) and 2 rural drug vendors (run by health assistants). The University of Gondar Referral and Teaching Hospital (UoGRTH) is the only referral and teaching center in the area with multiple specialized clinics, including pediatrics, surgery, gynecology, psychiatry, HIV care and an outpatient’s clinic; the hospital acts as the referral center for four district hospitals in the area and gives service to more than 5 million residents of Gondar and neighboring towns.

### Sampling and recruitment strategies

We surveyed three groups namely, patients, physicians and pharmacy professionals working in community drug retail outlets (CDROs). In the first survey group, a convenience sample of adult patients who attended UoGRTH outpatient clinic between January 1 and February 1, 2017 were invited to participate. Adult patients (at least 18 years of age) regardless of the type of diagnosis, who had at least one prescription and patients who gave their consent were eligible to be included in the study. Patients who had severe physical or psychological problems, which could lead to the inability to complete the questionnaire and those who refused to participate were excluded from the survey.

In the second survey group, it was anticipated that physicians could be approached via the Ethiopian Medical Association (EMA), a national professional organization that represents Ethiopian medical doctors. We approach physicians via EMA as it encompasses physicians working in various sectors including private and government hospitals, academicians as well as clinical researchers, which could give us a representative sample of prescribers. Accordingly, all physicians who attended 52^nd^ annual medical conference held in Addis Ababa in April 2016 were invited to participate. In the third survey group, all pharmacy professionals who were working in CDROs of Gondar town were our source population, while those pharmacy professionals who were available in the CDROs during the study period were our study population and approached to participate in the study. In all survey groups, questionnaires were distributed and respondents were requested to hand in the completed questionnaires. A final sample size of 400 patients, 201 physicians and 86 pharmacy professionals were invited to participate in the survey using convenient sampling method.

### Survey instrument

Data collection was performed by two of the authors (BMG, and DAE) through self-administered questionnaire and partly by explaining the questions for those who were unable to understand. The questionnaire was adopted by modifying items in previously used instruments regarding patients’ and physicians’ perceptions, as well as pharmacy professionals towards generic medicines [5, 27–30]; all items were thoroughly reviewed for relevance by the authors. The questionnaire, first prepared in English, was translated to Amharic language and back to English so as to ensure that the translated version gave the proper meaning. The survey instrument was further validated by pilot-testing on voluntary respondents (25 patients, 10 pharmacy professionals 10 physicians). After collecting feedback from the respondents, which were not included in the final survey, slight modification was instituted in the final data collection tool. The data collection tool included two main parts. Section one explored the socio-demographic characteristics of respondents including age, gender, marital status, educational level and work experience. The second section aimed to assess the perception of respondents regarding generic medicines using a five Likert scale response (Strongly Agree = 5, Agree = 4, Neutral = 3, Disagree = 2, and Strongly Disagree = 1).

### Statistical analysis

The data collected were entered into and analyzed using Statistical Package for the Social Sciences (SPSS) software version 21.0 for Windows (SPSS Inc., Chicago, IL). The survey questionnaire on the perception of generics was categorized into five Likert scale responses (Strongly Agree = 5, Agree = 4, Neutral = 3, Disagree = 2, and Strongly Disagree = 1). Frequencies, percentages and median were used to express different variables.

### Ethical considerations

The study was approved by the ethical review committee of School of Pharmacy at University of Gondar with a reference number of UoG-SoP/0152/17. Informed consent from all participants was gained prior to the commencement of the study. The data collected were kept anonymous and recorded in such a way that the participant could not be identified.

## Results

A total of 612 participants (391 patients, 143 physicians and 78 pharmacy professionals) completed the questionnaire, giving a response rate of 97.7%, 71.1% and 90.7% respectively.

### Patients’ perspective

More than half of the respondents (56%, n = 219) knew about the term brand and generic medicines and most of them pay full cost of their prescription themselves (60.6%, n = 237). The socio-demographic characteristics of respondents are shown in Table 1.

**Table 1:** Socio-demographic characteristics of patients, Gondar, 2017.

Variable	Frequency (%)
**Gender**	
Male	205 (52.4)
Female	186 (47.6)
**Occupation**	
Government employee	130 (33.2)
Private worker	78 (19.9)
Student	92 (23.5)
Farmer	61 (15.6)
House wife	30 (7.7)
**Monthly income (in USD)**	
<100	200 (51.1)
101–199	55 (14.1)
>200	136 (34.8)
**Educational status**	
Illiterate	70 (17.9)
Primary school	49 (12.5)
High school	191 (48.8)
College and above	81 (20.7)
**Percentage paid from the prescription**	
Pay full cost	237 (60.6)
All paid by family	78 (19.9)
All paid by government	52 (13.3)
Percentage paid by family	18 (4.6)
Percentage paid by government	6 (1.5)
**Know about brand/generic medicine**	
Yes	219 (56)
No	172 (44)
**Prescriptions are filled with**	
Generic named medicine	141 (36.1)
Brand named medicine	87 (22.3)
Both	163 (41.7)
**Chronic medical condition**	
Cardiovascular disorder	123 (31.5)
Respiratory disorder	74 (18.9)
Endocrine disorder	83 (21.2)
Other	111 (28.4)
**General health status**	
Poor	59 (15.1)
Fair	90 (230)
Good	171 (43.7)
Very good	50 (12.8)
Excellent	21 (5.4)

Patients’ perceptions about generic medicines are tabulated in Table 2. According to the survey, majority (57.5%, n = 225) of the respondents agreed/strongly agreed that the physician should ask them about their medicines preference and nearly half (47%, n = 184) of them agreed that they should have the option of choosing between generic and brand medicines. Furthermore, nearly half (49.4%, n = 193) of the respondents agreed/strongly agreed that brand name medicines are more effective than generics and they should be reserved for the treatment of serious diseases. In terms of quality, more than one-third (36.6%, n = 143) of the respondents agreed/strongly agreed that generic medicines are made with lower quality substances. More than two-thirds of respondents (75.2%, n = 294) accept the pharmacist substituting their prescribed medications to a cheaper medicine and similar percentage of patients (78%, n = 305) preferred to be prescribed locally produced medicines. Regarding cost considerations, the majority of respondents (83.1%, n = 325) agreed/strongly agreed that the costs of medicines are, in general, too high and more than three quarters (80%, n = 313) of the surveyed patients strongly disagreed/disagreed that the costs of medicines are not a major issue in the management of their medical condition.

**Table 2:** Patients’ perception about generic medicines, Gondar, 2017.

Survey questions	Frequency (%)			
	Disagree/strongly disagree	Neutral	Agree/Strongly agree	Median (IQR)
Physicians should ask patients about their medicines preference.	139 (35.5)	27 (6.9)	225 (57.5)	4 (2–4)
Patients should have the option of choosing between generics and brands	122 (31.2)	85 (21.7)	184 (47)	3 (2–4)
I don’t mind the pharmacist substituting the medicine I was prescribed to a cheaper equivalent one.	66 (16.9)	31 (7.9)	294 (75.2)	3 (2–4)
Generic medicines take longer time to be efficacious.	116 (29.6)	121 (31)	154 (39.4)	3 (2–4)
Generics are safer than brand name medicines.	139 (35.6)	143 (36.6)	109 (27.8)	3 (2–4)
Brand name medicines are more effective than generics.	93 (23.7)	105 (26.8)	193 (49.4)	3 (3–4)
Generic medicines are good for less serious diseases.	98 (25.1)	129 (33.0)	164 (41.9)	3 (2–4)
Generic medicines shouldn’t be used for serious diseases.	126 (32.2)	97 (24.8)	168 (43.0)	3 (2–3)
Generic medicines are made with lower quality substances.	131 (33.5)	117 (30.0)	143 (36.6)	3 (2–4)
I prefer to be prescribed locally produced medicines.	64 (16.4)	21 (5.4)	305 (78)	3 (2–4)
I prefer to be prescribed a well-known brand.	76 (19.4)	121 (30.9)	194 (49.7)	3 (3–4)
I prefer to be prescribed imported rather than local medicines.	114 (29.1)	116 (29.7)	161 (41.1)	3 (2–4)
Costs should be considered before a drug is prescribed.	101 (25.8)	50 (12.8)	240 (61.4)	4 (2–5)
I don’t mind whether my prescribed/dispensed medicine is locally produced or imported as long as it is effective.	140 (35.8)	96 (24.6)	155 (39.7)	3 (2–4)
I prefer to be prescribed/dispensed the cheapest medicine available for the treatment of my condition.	128 (32.8)	65 (16.7)	198 (50.6)	4 (2–4)
Cost is not an issue for me as long as the medicine will treat my condition.	313 (80)	20 (5.1)	58 (14.7)	3 (2–4)
A more expensive medicine is a better one.	186 (47.5)	77 (19.7)	128 (32.8)	3 (2–4)
Imported medicines are better.	122 (31.2)	82 (21.0)	187 (47.8)	3 (2–4)
Using generic medicines would provide significant saving to me.	107 (27.3)	138 (35.3)	146 (37.4)	3 (2–4)
In general, medicine costs in Ethiopia are too high.	48 (12.3)	18 (4.6)	325 (83.1)	4 (3–4)

IQR: Inter quartile range

### Physicians’ and pharmacy professionals’ perspective

The demographic characteristics and perceptions of physicians and pharmacy professionals towards generic medicines are tabulated in Tables 3 and 4. The majority of physicians (70.6%, n = 101) indicated a very low generic medicine prescription rate. According to the survey, majority (91.9%, n = 130) of physicians and pharmacy professionals (87.2%, n = 68) agreed/strongly agreed that they need a standard guideline to both prescribers and pharmacists on brand substitution processes. Most physicians (93.7%, n = 134) and pharmacy professionals (91.0%, n = 71) agreed to the notion that collaboration between physicians and pharmacy professionals would boost patients’ quality use of generic medicines. A similar percentage of physicians and pharmacy professionals (88.1%, n = 126 and 89.7%, n = 70 respectively) agreed that patient should be given enough information about generic medicines in order to make sure they really understand about their medicines. Furthermore, the majority of physicians (83.2%, n = 119) and pharmacy professionals (92.3%, n = 72) expressed a need for more information on the issues pertaining to the safety and efficacy of generic medicines. It is also interesting to note that only 39.9%, n = 57 of the physicians agreed/strongly agreed that drug advertisements and promotion by the manufacturers would influence their prescribing practice, while the majority of surveyed pharmacy professionals (87.2%, n = 68) agreed that drug advertisements would influence their dispensing practice. Finally, when asked about the influence of drug procurement budget, majority of the physicians (89.5%, n = 128) and pharmacy professionals (59%, n = 46) agreed/strongly agreed that it would affect their choice of medicine (Tables 3 and 4).

**Table 3:** Demographic characteristic of surveyed physicians and pharmacy professionals, 2017.

Variable	Frequency (%)	
	Physicians, n = 143	Pharmacy professionals, n = 78
**Gender**		
Male	82 (57.3%)	41 (52.6%)
Female	61 (42.7%)	37 (47.4%)
**Age (years)**		
<30	31 (21.7%)	45 (57.7%)
30–40	50 (35%)	21 (26.9%)
41–50	33 (23.1%)	8 (10.2%)
>50	29 (20.2%)	4 (5.1%)
**Post graduate degree**		
Yes	79 (55.2%)	17 (21.8%)
No	64 (44.8%)	61 (78.2%)
**Work experience**		
<5 years	51 (35.7%)	34 (43.6%)
6–10 years	33 (23.1%)	29 (37.2%)
>11 years	59 (41.2%)	15 (19.2%)
**No. of prescription written or dispensed per day**		
<50	48 (33.6%)	11 (14.1%)
51–100	74 (51.7%)	15 (19.2%)
>100	21 (14.7%)	52 (66.7%)

**Table 4:** Health care provider’s (physicians, pharmacy professionals) perceptions regarding generic medicines, 2017.

Survey question			I believe we need a standard guideline to both prescribers and pharmacist on brand substitution process.	In my opinion, quality use of generic medicines among patients can be achieved if both prescribers and pharmacist work together.	I think patient should be given an enough information about generic medicines in order to make sure they really understand about the medicines they take.	I believe advertisement by the drug companies will influence my future prescribing/dispensing pattern.	I need more information on the issues pertaining to the safety and efficacy of generic medicines.	Hospital budget for drug procurement factor will affect my choice of medicines.
**Frequency (%)**	Agree/Strongly agree	Physicians	130 (91.9%)	134 (93.7%)	126 (88.1%)	57 (39.9%)	119 (83.2%)	128 (89.5%)
		Pharmacy professionals	68 (87.2%)	71 (91.0%)	70 (89.7%)	68 (87.2%)	72 (92.3%)	46 (59.0%)
	Neutral	Physicians	4 (2.8%)	1 (0.7%)	8 (5.6%)	10 (7%)	9 (6.3%)	6 (4.2%)
		Pharmacy professionals	4 (5.1%)	2 (2.6%)	1 (1.3%)	5 (5.1%)	0 (0%)	12 (15.4%)
	Disagree/strongly disagree	Physicians	9 (6.3%)	8 (5.6%)	9 (6.3%)	76 (53.1%)	15 (10.5%)	9 (6.3%)
		Pharmacy professionals	6 (7.7%)	5 (6.4%)	7 (9%)	5 (5.1%)	6 (7.7%)	20 (25.6%)

## Discussion

Generic medicines are an indispensable part of Ethiopia’s drug expenditure management strategy. Exploring the perceptions of various stakeholders, including patients, physicians and pharmacy professionals about generic medicines has implications for promoting the quality use of equally effective generic medicines and establishing a comprehensive generic medicine policy in the country [31]. To the best of our knowledge, this is the first attempt to investigate the perceptions of patients, physicians and pharmacy professionals about generic medicines in Ethiopia.

According to the findings from this survey, patients generally tend to accept generic medicine substitutions. Similar findings were reported in previous studies done in south Africa [32, 33]. It is also interesting to note that more than three quarters (75.2%, n = 294) of respondents in our study accept the pharmacist substituting their prescribed medications to a cheaper medicine and preferred to be prescribed imported, rather than local, medicines. In a similar study conducted in Jordan, about 78% of respondents accepted generic substitution [28]. The high tendency to accept generic substitution in our study could be partially explained by the majority patients could not afford to purchase branded medicines, as most patients covered their medical costs themselves, mainly out of pocket money [26]. This notion is further strengthened by the idea that many patients may not communicate adequately with their physicians regarding medication-related decisions.

In terms of quality and efficacy, a significant proportion of respondents agreed that brand name medicines are more effective than generic medicines and agreed that generic medicines are made with lower quality substances; this is an issue which warrants special attention. This negative view could be partially attributed to the fact that generic medicines produced locally are of low quality compared to the imported brand medications in terms of safety and efficacy. In 2012, the Food, Medicine, Healthcare Authority and Control Agency (FMHACA) of Ethiopia adopted the five-year good manufacturing practice (GMP) Roadmap (2013–2018) in collaboration with the United States Pharmacopeia/Promoting the Quality of Medicines (USP/PQM), the WHO and local manufacturers which aimed at improving access to, affordable, safe, and good-quality medicines produced in Ethiopia. From assessment of eight Ethiopian pharmaceutical companies, six of them fells to level 1 out of the 3 levels (manufacturers with up to 50% GMP compliance) [34]. To better ensure the quality of medicines, the government of Ethiopia recently developed a decade long new strategic plan for pharmaceutical manufacturing [35], with the objective of improving access to affordable and effective medicines through supporting and advocating the local production of pharmaceuticals. Ethiopia is currently developing an incentive that strengthens local medicine manufacturers so as to reduce the higher price of imported brand medications and to ultimately make sure patients get the health care in the most cost-effective way. However, the issue of quality and price of medicines in Ethiopia, either locally manufactured or imported, should be the core agenda for the governments’ new strategic plan in the pharmaceutical sector.

It is also interesting to note that more than three quarters (83.1%, n = 325) of respondents agreed or strongly agreed that the costs of medicines in general are too high and reported this as a major issue in the management of their medical condition. Similarly, the majority of surveyed physicians and pharmacy professionals agreed that drug procurement budgets would affect their choice of medicine. This could, in turn, affect patients’ adherence to therapeutic regimens [36]. In Ethiopia, although the per capita health expenditure has increased from US $7.14, in 2005, to US $16.1, in 2008, a significant number of patients still depend on out of pocket money as the main sources of drug expenditure; this accounts for approximately 37% of the total health expenditure [26, 37, 38]. This was further supported by the finding of our study, where 80% of respondents either paid full or part costs of their prescribed medicines. Taking into account the patients inability to purchase more expensive brand medications along with their preferences to be prescribed and/or dispensed the cheapest medicine available for the treatment of their medical condition, physicians and pharmacy professionals should consider the cost and affordability by the patient while prescribing or dispensing medicines.

Many physicians opposed brand substitution, believing that generic medicines were inferior to their brand equivalents [39]. This notion was further supported by the findings of our survey, in which the majority of surveyed physicians indicated a very low generic medicine prescription rate. Similar findings have also been reported in Malaysia [29]. Similarly, the majority of surveyed pharmacy professionals often dispense brand medications upon patient request, rather than generic medicines. The physicians’ and pharmacy professionals’ low rate of prescribing and dispensing of generic medicines are further influenced by drug advertising and promotion, as evidenced by the findings of this survey, where 39.9% of the physicians and 87.2% of the pharmacy professionals agreed/strongly agreed that advertisement by the drug companies influences their prescribing/dispensing pattern. Similarly, a study done in Sudan reported that 53.8% of physicians and 44.6% of pharmacy professionals were moderately influenced by pharmaceutical promotion [40]. Furthermore, 91% of physicians and 87.2% of pharmacy professionals expressed the need for a standard guideline on generic substitution. The enactment of such guidelines would increase the use of generic medicines and boost its accessibility and affordability [41]. Generic substitution guidelines allow the pharmacy professionals to dispense a different brand of the drug, even when the prescription is written for another branded medicine [42]. However, such practice is not currently supported by law in Ethiopia [43]. Furthermore, the majority of physicians and pharmacy professionals agreed/strongly agreed that it was crucial to establish collaboration between physicians and pharmacy professionals in order to improve generic medicine use. They also advocate the need to endow patients with more information about generic medicines. The findings of the present survey call for customized educational intervention programs to improve the perception of patients and healthcare professionals (mainly pharmacy professionals and physicians) to enhance the utilization of generic medicines, which has been proven to be an effective strategy in other settings [44].

### Limitation of the study

This survey highlights an area of practice where there is lack of literature in Ethiopia. Yet, the survey has some limitations that should be taken into account while interpreting the results. As the study was a descriptive survey conducted in only Gondar and utilizes a convenience sampling technique, caution should be exercised when generalizing to other cities and regions in Ethiopia. Moreover, the use of a self-administered questionnaire, which depends on honesty and faith of the respondents, could affect the responses as it may be subjected to response or recall bias. Even with the above limitations, this survey has significant implications for improving the practice of generic prescribing and/or substitution in Ethiopia.

## Conclusion

Overall, our findings demonstrate a knowledge gap among patients towards the perception of generic medicines, perceiving generics as less effective and inferior in quality compared to their branded equivalents. The majority of physicians reported a very low generic medicine prescription rate and the majority of pharmacy professionals’ dispensing practice were influenced by drug advertisements. Hence, a customized educational program could be developed and implemented to patients, prescribers and dispensers so as to boost the acceptability of generic medicines and increase generic prescribing and/or substitution. Furthermore, implementation of national policy on generic substitution and medicine pricing is recommended to promote the use of generics and reduce the drug expenditure and improve the medicine affordability.

## Abbreviations

CDROs: Community drug retail outlets
EMA: Ethiopian Medical Association
SPSS: Statistical Package for the Social Sciences
USD: United States Dollar
UOGRTH: University of Gondar Referral and Teaching Hospital
WHO: World Health Organization
